# Quantitative Assessment of Airway Changes in Fibrotic Interstitial Lung Abnormality Patients by Chest CT According to Cumulative Cigarette Smoking

**DOI:** 10.3390/tomography8020082

**Published:** 2022-04-03

**Authors:** Yuan Zhe Li, Gong Yong Jin, Kum Ju Chae, Young Min Han

**Affiliations:** 1 Department of Radiology, Jeonbuk National University Medical School, Jeonju 54896, Korea; lyzhe1992@gmail.com (Y.Z.L.); para2727@gmail.com (K.J.C.); ymhan@jbnu.ac.kr (Y.M.H.); 2 Research Institute of Clinical Medicine, Biomedical Research Institute of Jeonbuk National University Hospital, Jeonbuk National University Medical School, Institute of Medical Science, Jeonju 54970, Korea

**Keywords:** smoking, bronchi, diagnosis, computer-assisted

## Abstract

Purpose: The aim of this study was to evaluate the role of Pi10 in patients with fibrotic interstitial lung abnormality (fibrotic ILA) in a chest CT, according to cumulative cigarette smoking. Methods: We retrospectively assessed 54 fibrotic ILA patients and 18 healthy non-smokers (control) who underwent non-enhanced CT and pulmonary function tests. We quantitatively analyzed airway changes (the inner luminal area, airway inner parameter, airway wall thickness, Pi10, skewness, and kurtosis) in the chest CT of fibrotic ILA patients, and the fibrotic ILA patients were categorized into groups based on pack-years: light, moderate, heavy. Airway change data and pulmonary function tests among the three groups of fibrotic ILA patients were compared with those of the control group by one-way ANOVA. Results: Mean skewness (2.58 ± 0.36) and kurtosis (7.64 ± 2.36) in the control group were significantly different from those of the fibrotic ILA patients (1.89 ± 0.37 and 3.62 ± 1.70, respectively, *p* < 0.001). In fibrotic ILA group, only heavy smokers had significantly increased Pi10 (mean increase 0.04, *p* = 0.013), increased airway wall thickness of the segmental bronchi (mean increase 0.06 mm, *p* = 0.005), and decreased lung diffusing capacity for carbon monoxide (*p* = 0.023). Conclusion: Pi10, as a biomaker of quantitative CT in fibrotic ILA patients, can reveal that smoking affects airway remodeling.

## 1. Introduction

There has been increased awareness of incidentally detected interstitial lung abnormalities (ILAs) in smokers and participants of lung cancer screening trials, and fibrotic ILA on chest CT has been shown to progress radiologically within 2–4 years of follow-up [1,2,3,4]. ILA could be defined as non-dependent changes affecting more than 5% of any lung zone, including non-dependent ground-glass or reticular abnormalities, nonemphysematous cysts, honeycombing, and traction bronchiectasis [1,2,5]. It has been recognized that ILAs are associated with poor clinical outcomes and an increased rate of mortality, and the rate of imaging progression ranges from 20% over 2 years to 63% over 5 years [1,2,3].

The presence of ILA is associated with decreased total lung capacity and individuals with ILA also showed impaired gas exchange compared with those without ILA [5,6,7,8]. The association between ILA and spirometry results, however, is controversial because some of these differences may be affected by older age and smoking history, which are common in both ILA and decline in spirometry values [5]. Since 10 years ago, given the limitations of spirometry in the characterization of chronic obstructive pulmonary disease (COPD), quantitative computed tomography (QCT) is increasingly being used to evaluate different COPD phenotypes, such as emphysema, large airway remodeling, and expiratory air trapping [8,9,10,11,12]. There are few papers on the quantification of ILA found on chest CT. Miller et al. [13] also reported that in COPD Gene and ECLIPSE, research participants (more than 30 pack-years) with ILA had increased measures of Pi10 compared with those without ILA. What is clear is that Pi10 is possible to increase when ILA on the Chest CT is found in a patient with 30 pack-years of heavy smoking [13]. However, there are few studies on Pi10 when ILA was found on chest CT in less than 30 pack-years of a smoker because existing studies have selected and analyzed ILA subjects from data of lung scanner screening targeting 30 pack-years of heavy smoking. In addition, there is no date for quantitative analyses investigating which parameters assessed by pulmonary function tests are affected by pack-years in fibrotic ILA patients.

As mentioned above, spirometry is known to be affected by smoking in ILA patients, but how QCT parameters change to be affected by smoking in fibrotic ILA patients is unknown. In this study, we conducted QCT measurements (airway and lung density) and compared the results with pulmonary function test results in fibrotic ILA patients according to cumulative cigarette smoking. We investigated that smoking pack-years would affect airway remodeling and change of lung density in fibrotic ILA patients and that the degree of these alterations would associate with pulmonary function test results.

## 2. Materials and Methods

### 2.1. Study Subjects

This was a retrospective study approved by our Institutional Review Board. Therefore, informed consent was waived. We obtained medical records and non-enhanced CT images taken from September 2013 to October 2015, which were reviewed by a radiologist. We included smoking male patients over 50 years old diagnosed with ILA, emphysema, or interstitial pneumonia. Since the QCT parameter can be affected by gender, only male smokers were limited [11].

Diagnosis of fibrotic ILA is confirmed on the basis of findings of chest CT as follows [14]: (1) incidental identification of non-dependent abnormalities, including ground-glass with reticular abnormalities, lung distortion, traction bronchiectasis, honeycombing, and non-emphysematous cysts; (2) involving at least 5% of a lung zone (upper, middle, and lower lung zones are demarcated by the levels of the inferior aortic arch and right inferior pulmonary vein): (3) in individuals in whom interstitial lung disease is not suspected.

There were 75 patients with PFTs performed on the same day as the chest CT scans. Among them, 21 patients were excluded because they matched at least one of the following exclusion criteria: idiopathic non-specific interstitial pneumonia (NSIP) without emphysema because ground-glass without reticular abnormalities, lung distortion, traction bronchiectasis, honeycombing (*n* = 3), the presence of connective tissue disease (*n* = 3), bronchopneumonia (*n* = 5), pneumoconiosis (*n* = 1), active pulmonary tuberculosis (*n* = 2), lung malignancy (*n* = 4) or image noise that prevented image analysis (*n* = 3). Thus, 54 fibrotic ILA patients were included in the study.

**Figure 1:**
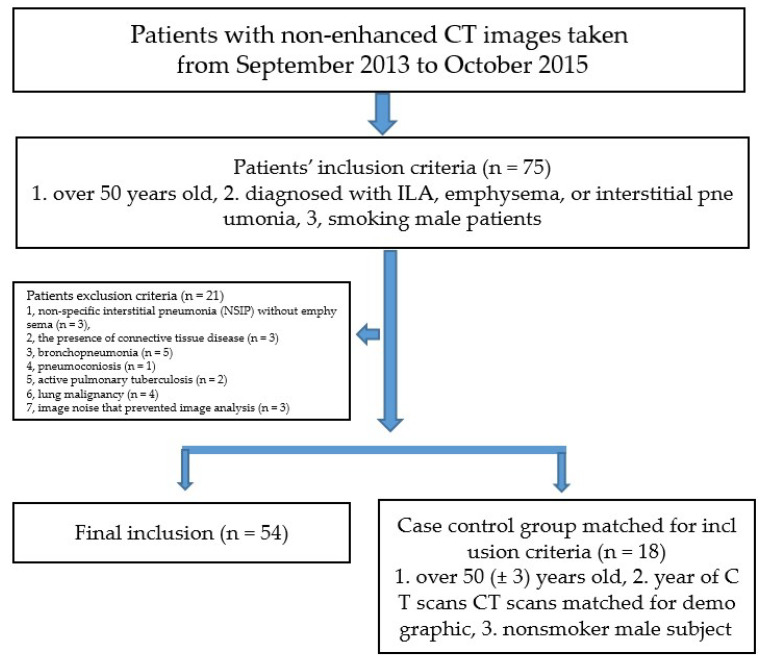
Flow chart of the study population.

A matching process was used to identify controls in order to reduce confounding effects. Control subjects matched for demographic variables including age (±3 years), year of CT scans, and smoking status (nonsmoker) were used as controls. A total of 18 subjects were enrolled as controls (Figure 1).

### 2.2. Pulmonary Function Tests

PFTs were performed according to the American Thoracic Society guidelines [15]. A portable spirometer (Chest Graph HI-701, Chest Co., Ltd., Tokyo, Japan) was used, and the following values were assessed: FEV_1_, forced vital capacity (FVC), FEV_1_/ FVC ratio, and diffusion capacity of the lung for carbon monoxide (DLco). All PFTs were performed on the same day as the chest CT scans.

### 2.3. Smoking

Status of smoking exposure was classified as: light smoker (≤10 cigarettes daily), moderate smoker (≤20 cigarettes daily), and heavy smoker (>20 cigarettes daily). Pack-years of smoking were calculated by multiplying the number of years smoked by the average number of packs per day. Non-smoker patients were the control [16].

### 2.4. CT Acquisition

A multi-detector CT scanner (Somatom Sensation 16, Siemens Medical Solution, Erlangen, Germany, or Somatom Definition FLISH, Siemens Medical Solution, Forchheim, Germany) was used to take the volumetric assessment. The patients were required to hold their breath with deep inspiration in the supine position. The following CT parameters were used: tube current of 200 mAs, tube voltage of 120 kVp, rotation time of 0.5 or 0.33 s, reconstructed slice thickness of 1.0 mm, reconstructed slice interval of 0.7 mm, reconstruction kernel of B35f, and acquisition of 16 × 1 or 128 × 0.6 mm.

### 2.5. Quantitative CT Evaluation

All CT images were quantitatively assessed using Pulmonary Workstation using the APOLLO software (VIDA Diagnostics, Inc., Coralville, IA, USA). Airway segmentations were assessed to ensure consistent branching structure across all acquired segmentations with the merging of fractured branches occurring when required. The airway tree was automatically generated using the region growing technique. All quantitative airway data were automatically collected from the inspiration CT and included the inner luminal area (iLA), airway inner parameter (AIP), airway wall thickness (AWT), and wall area fraction (WAF; percentage wall area/total bronchial area). The Pi10 is derived by plotting the square root of the airway wall area against the internal perimeter of each measured airway and using the regression line to calculate the square root of the airway wall area for a hypothetical representative airway with an internal perimeter of 10 mm [17]. The Pi10 value was obtained as a global comparative measure using 6 segmented airway branches.

The extent of emphysematous lesions was determined by evaluating the low attenuation area (values lower than −950 Hounsfield, %LAAI-950), the threshold value at the 15th percentile (PER15, HU), and mean lung attenuation (MLA%) [18,19]. Total lung capacity (TLCCT) was also calculated based on inspiratory CT images.

### 2.6. Statistical Analysis

All descriptive data are depicted as the mean and standard deviation (SD) for the continuous variables. Independent sample t-tests and one-way ANOVA analyses were used to compare normally distributed continuous variables. The Mann–Whitney U test and Kruskal–Wallis test were used for non-normally distributed data. Statistical analyses were performed using SPSS 12.0.1 software. Associations were considered statistically significant at *p* < 0.05.

## 3. Results

PFT results and the measured QCT parameters of fibrotic ILA patients and healthy control subjects are listed in Table 1. The mean age was 68.56 ± 6.03 years in the control group and 70.51 ± 7.51 years in the fibrotic ILA group. The DLco was significantly different between the two groups, with the fibrotic ILA group demonstrating a much lower diffusion capacity (72.90 ± 21.48%) compared to controls (110.00 ± 9.56%) (*p* < 0.001) regardless of smoking pack-years. Additionally, the LAAI-950 was significantly different between the control (0.64 ± 0.58%) and fibrotic ILA groups (5.39 ± 6.40 %) (*p* < 0.001). Mean skewness (2.58 ± 0.36) and kurtosis (7.64 ± 2.36) in the control group were significantly different from those of the fibrotic ILA patients (1.89 ± 0.37 and 3.62 ± 1.70, respectively, *p* < 0.001). However, airway QCT parameters such as AIP, AWT, WAF, and Pi10 showed no difference between control and fibrotic ILA groups.

**Figure 2:**
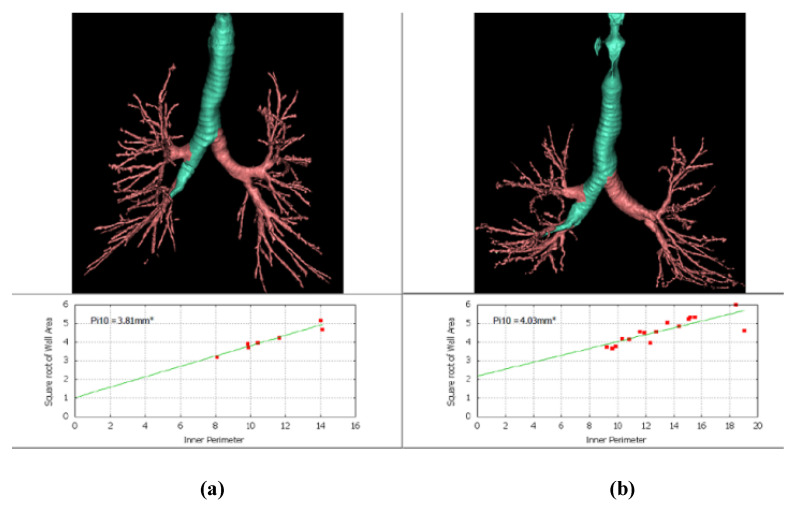
Quantitative data of Pi10 in the right middle lobe in two patients: (**a**) A 78-year-old male in the moderate smoker group (Pi10 = 3.81 mm^2^) and (**b**) an 82-year-old male in the heavy smoker group (Pi10 = 4.03 mm^2^).

**Figure 3:**
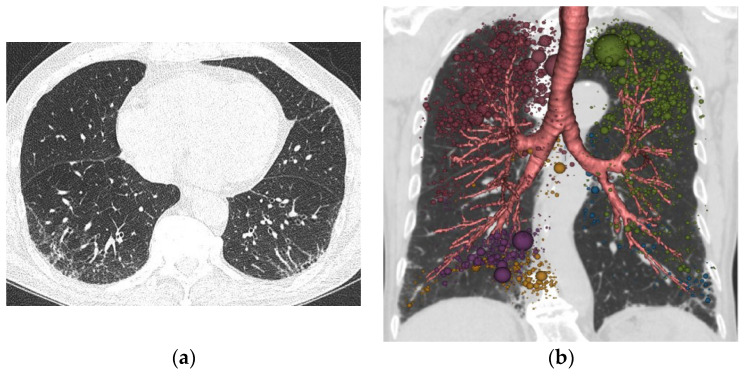
(**a**) 78-year-old man who belonged to the moderate smoking group. A. Chest CT shows ground-glass opacity with reticular opacity in predominantly subpleural area of posterobasal segment, both lower lobe; (**b**) Quantitative data from chest CT shows upper lobe predominant emphysema with a quantified low attenuation area (LAA_I-950_: 1.62%) and Pi10 (3.81 mm).

**Figure 4:**
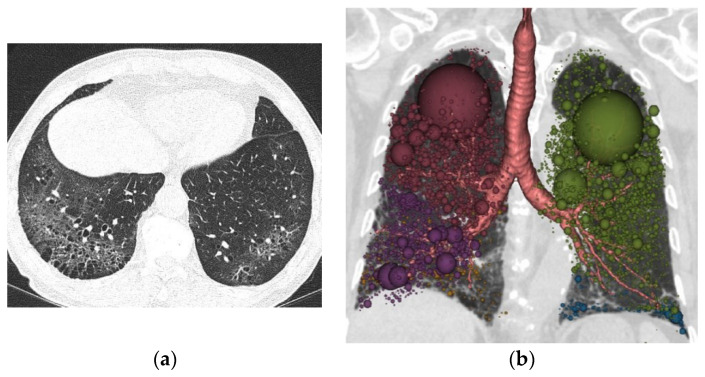
(**a**) 82-year-old man who belonged to the heavy smoking group. A. Chest CT shows ground-glass opacity with honeycomb in predominantly subpleural area of posterobasal segment, both lower lobe; (**b**) Quantitative data from chest CT shows upper lobe predominant emphysema with a quantified low attenuation area (LAA_I-950_: 10.97%) and Pi10 (4.03 mm^2^).

A comparison of QCT and PFT measurements according to pack-years is shown in Table 2. Of the PFT measurements, only the DLco was significantly different among the three groups, and the heavy smoker group had severely decreased DLco (mean, 58.36 ± 11.83 %). Meanwhile, MLA, kurtosis, AWT, and Pi10 were significantly different in the QCT measurements among fibrotic ILA groups assessed (Figure 2). When comparing airway parameters at the segmental level according to pack-years of smoking, the iLA, AIP, and WAF results were not significantly different among the three groups. However, the AWT results were significantly different among the three groups (*p* = 0.005). The mean AWT was 1.77 ± 0.11 in the control group, 1.71 ± 0.09 in light, 1.72 ± 0.08 in moderate, and 1.83 ± 0.14 in heavy. The mean Pi10 was 3.97 ± 0.05 in the control group, 3.96 ± 0.07 in light, 3.96 ± 0.05 in moderate, (Figure 3), and 4.01 ± 0.05 in heavy (Figure 4), (*p* = 0.013).

Definition of abbreviations: FVC, forced expiratory vital capacity; FEV_1_, forced expiratory volume in 1 s; FEF25–75%, forced expiratory flow between 25–75%; DLCO, diffusion capacity of lung for carbon monoxide; TLC, total lung capacity; Pi10, inner perimeter of 10 mm; MLA, mean lung attenuation; %LAAI-950, percentage area with CT attenuation values less than −950 HU at inspiration; LA, inner luminal area of segmental bronchi; AWT, airway wall thickness of segmental bronchi; AIP, airway inner parameter; WAF, wall area fraction of segmental bronchi (percentage wall area/total bronchial area).

Control: non-smoking patients, light: ≤10 cigarettes daily, moderate: ≤20 cigarettes daily, heavy: >20 cigarettes daily.

## 4. Discussion

In this study, we demonstrated that smoking pack-years in fibrotic ILA male patients was significantly associated with the Pi10 and DLco. In more detail, increased Pi10 and decreased DLco in fibrotic ILA occurred in patients with only heavy smoking. Pi10 is not different between fibrotic ILA in less heavy smokers and normal subjects and this result could explain why FEV_1_ often has a limited role in the diagnosis of fibrotic ILA. Therefore, a quantitative measurement of airway remodeling such as Pi10 may play a role in evaluating smoking-related airway alteration of fibrotic ILA according to cumulative cigarette smoking. Additionally, it is thought that by predicting information about DLco through the results of smoking pack-years with Pi10 values, skew, and kurtosis on QCT on CT of a heavy smoker, the subject may be able to help with further examination of PFT.

Pi10 is defined as the airway wall thickness at an internal perimeter of 10 mm and is used to measure the overall airway wall thickness of the lung. For each airway, manual tracings are obtained of the total airway (Ao) and the luminal airway (Ai), and the AWT is calculated as the result of Ao–Ai. Pi10 is then assessed from the slope of the regression line calculated by comparing the square root of the wall area measures plotted against the internal perimeter of the airway [13]. The increase of Pi10 in obstructive airway diseases such as COPD is well known, in which increased Pi10 measures have been associated with worsening respiratory symptoms, increased frequency of COPD exacerbations, and increased mortality risk [18,19]. The median value for Pi10 commonly reported in patients with COPD and biopsy-proven IPF was 3.69–4.94 mm and 5.78 mm [20,21]. In our study, the median value for Pi10 of fibrotic ILA was 3.96–4.01 mm. Increased Pi10 values have been associated with chronic bronchitis (0.03 mm), bronchodilator responsiveness (0.04 mm), and increases in respiratory symptoms (0.1 mm) in patients with COPD compared to those without COPD [17,22,23]. In our study, the median value for Pi10 noted in fibrotic ILA patients with heavy smoking is 0.05 mm larger than non-smokers and in patients with light and moderate smoking.

The value of Pi10 is still ambiguous because of covariates such as age, sex, race, body mass index, smoking behavior, and chronic obstructive pulmonary disease severity when appropriate [13]. Kim et al. [11] reported that the Pi10 value in normal Korean male subjects is significantly greater than that in normal Korean female subjects in spite of normal spirometry. In Korean subjects with normal spirometry and a visually normal chest CT, there may be significant differences in Pi10 according to sex, age, and smoking history. However, what is meaningful is that Pi10 in ever-smokers was significantly correlated with forced expiratory volume in 1 s/forced vital capacity (r = −0.455, *p* = 0.003). When the relationship between Pi10 and FEV_1_ was investigated for men who participated in the Dutch–Belgian Lung Cancer Screening Trial (NELSON) study, participants with a higher Pi10 had a lower FEV_1_ (r = −0.49 and 0.11, respectively, *p* < 0.001) [24,25,26]. Additionally, Miller et al [13] reported that after adjustment for important covariates (e.g., age, sex, race, etc.), research participants with ILA had increased measures of Pi10 compared with those without ILA (0.03 mm in COPDGene, 95% confidence interval [CI], 0.02–0.03; *p* = 0.001; 0.02 mm in ECLIPSE, 95% CI, 0.005–0.04; *p* = 0.01; 0.07 mm in FHS, 95% CI, 0.01–0.1; *p* = 0.01) Our study revealed that the Pi10 increased as pack-years increased, suggesting that airway remodeling had occurred although it was not correlated with the spirometry results. In our study, airway remodeling only occurred in fibrotic ILA patients with heavy smoking, which indicates that only heavy pack-years play an important role in the airway changes in fibrotic ILA.

Although the FEV_1_ results of fibrotic ILA patients are controversial, FEV_1_ are often reduced. As mentioned above, it is argued that Pi10 and FEV_1_ in COPD and ILA patients have a negative correlation [8,11,13,24]. However, in our study, FEV_1_ showed no decrease in both normal and fibrotic ILA patients, although the number of subjects was small. Therefore, further research is needed on whether to perform PFT if there is no respiratory symptoms but there is a finding of fibrotic ILA on the chest CT. However, if you have a smoking history of more than 30 pack-years, are accompanied by fibrotic ILA on the chest CT, and Pi10 is abnormally increased, it is considered necessary to perform additional PFT to check for lung dysfunction. In previous studies [25], skewness and kurtosis are both correlated with DLCO. Additionally, Pi10 previously correlated with the DLco in COPD patients, although the extent of fibrotic lesions in the fibrotic ILA patients may introduce variability to the measurement of DLco [26]. In our study, Pi10 revealed a negative association with the DLco and this finding is in line with a previous report [27]. Therefore, we speculate that the increased Pi10, increased kurtosis, and decreased skew in QCT on chest CT may also associate with reduced DLco in fibrotic ILA patients with heavy smoking compared with never-smokers.

There are some limitations to our study. First, the pack-years and smoking status were retrospectively acquired, which may have caused information bias. Second, for Pi10 measures in patients with fibrotic ILA patients, we recommend caution in the interpretation of these findings. Although it is suggested that there is the possibility of true-negative associations between Pi10 and DLco, it is also possible that smaller sample sizes limited measurement variability. Therefore, future longitudinal assessments of Pi10 would be needed. Third, the extent of interstitial lesions in each patient has not been assessed in this study. Therefore, it could not be completely excluded that the extent of fibrosis due to ILA affects the measurement of Pi10.

## 5. Conclusions

In conclusion, in this study, the median value for Pi10 noted in fibrotic ILA patients with heavy smoking is larger than those of never-smokers. Therefore, a quantitative measurement such as Pi10 on chest CT may play a role in evaluating airway alteration of fibrotic ILA according to cumulative cigarette smoking. Additionally, we found that increased Pi10, as measured by QCT, and decreased DLco occurred often in fibrotic ILA patients with only heavy smoking. In addition, increased kurtosis, and decreased skew in QCT on chest CT may also be associated with reduced DLco in fibrotic ILA patients with heavy smoking compared with never-smokers. Then, it is thought that by predicting information about DLco through Pi10 values, skew, and kurtosis on QCT on chest CT of a heavy smoker, the subject may be able to help with further examination of PFT. Thus, Pi10 measured by QCT would be helpful to estimate airway remodeling of fibrotic ILA, especially in patients who are heavy smokers. However, future longitudinal assessments of Pi10 would be needed.

## Figures and Tables

**Table 1:** Compared Control Subjects with Fibrotic Interstitial Lung Abnormality Patients in Pulmonary Function Tests and Quantitative CT measurements.

Parameters	Control (*n* = 18)	Fibrotic ILA (*n* = 54)	*p* Value
Age(year) *	68.56 ± 6.03	70.51 ± 1.51	0.123
BMI(kg/m2) *	24.32 ± 2.78	23.40 ± 3.22	0.329
FVC (%)	95.89 ± 11.94	95.97 ± 15.32	0.983
FEV_1_ (%) *	105.89 ± 16.61	100.19 ± 18.79	0.439
FEV_1_/FVC *	77.00 ± 5.29	72.51 ± 9.97	0.070
FEF_25–75%_ (%) *	91.00 ± 26.87	80.94 ± 35.45	0.161
DLCO (%)	110.00 ± 9.56	72.90 ± 21.48	<0.001
TLCCT(L)	5146.77 ± 824.47	4909.71 ± 875.84	0.317
AIP (mm)	29.23 ± 2.62	29.96 ± 3.31	0.528
AWT (mm)	1.77 ± 0.11	1.79 ± 0.14	0.296
WAF (%)	0.49 ± 0.02	0.49 ± 0.02	0.439
Pi10(mm)	3.97 ± 0.05	3.98 ± 0.06	0.192
MLA(HU)	−839.86 ± 15.95	−823.64 ± 28.23	0.004
LAA_I-950_(HU) *	0.64 ± 0.58	5.39 ± 6.40	<0.001
Skewness *	2.58 ± 0.36	1.89 ± 0.37	<0.001
Kurtosis *	7.64 ± 2.36	3.62 ± 1.70	<0.001

Definition of abbreviations: BMI, Body mass index; FVC, Forced expiratory vital capacity; FEV_1_, Forced expiratory volume in 1 s; FEF25–75%, Forced expiratory flow between 25–75%; DLCO, Diffusion capacity of lung for carbon monoxide; TLC, Total lung capacity; AIP means airway inner parameter; AWT means airway wall thickness; WAF means wall area fraction; MLA, Mean lung attenuation; %LAAI-950, Percentage area with CT attenuation values less than −950 HU at inspiration. * non-normally distributed data which were used Mann–Whitney U test.

**Table 2:** Comparison of the Quantitative CT Measurements and Pulmonary Function Tests by Smoking Intensity in Fibrotic ILA patients.

Parameters	Control (*n* = 18)	Fibrotic ILA Patients (*n* = 54)				
		Light (*n* = 12)	Moderate (*n* = 24)	Heavy (*n* = 18)	*p*-Value	*Post hoc*
FVC (%)	95.89 ± 11.94	93.67 ± 19.21	96.76 ± 12.81	96.46 ± 16.32	0.933	
FEV_1_ (%)	105.89 ± 16.61	101.00 ± 25.89	97.74 ± 17.82	102.92 ± 14.87	0.406	
FVE_1_/FVC	77.00 ± 5.29	73.33 ± 12.09	70.06 ± 10.86	75.24 ± 6.21	0.080	
FEF_25–75%_ (%)	91.00 ± 26.87	87.92 ± 47.85	70.87 ± 26.75	90.19 ± 34.26	0.139	
DLCO (%)	110.00 ± 9.56	80.45 ± 24.45	77.41 ± 20.69	58.36 ± 11.83	<0.001	I, II III, IV
TLC_CT_(L)	5146.77 ± 824.47	4972.09 ± 1076.39	5031.03 ± 864.51	4706.35 ± 750.56	0.342	
MLA (HU)	−839.86 ± 15.95	−837.54 ± 25.31	−826.00 ± 28.80	−811.22 ± 25.30	0.004	I II III, IV
LAA_I-950_(HU) *	0.64 ± 0.58	6.10 ± 5.20	6.55 ± 8.19	3.38 ± 3.60	0.006	I IV, II III
AIP (mm)	29.23 ± 2.62	30.18 ± 2.32	29.42 ± 2.80	29.99 ± 3.04	0.867	
AWT (mm)	1.77 ± 0.11	1.71 ± 0.09	1.72 ± 0.08	1.83 ± 0.14	0.012	II III, IV
WAF (%)	0.49 ± 0.02	0.48 ± 0.02	0.49 ± 0.03	0.50 ± 0.02	0.146	
Skewness *	2.58 ± 0.36	1.94 ± 0.54	1.90 ± 0.33	1.82 ± 0.28	0.057	I IV, II III
Kurtosis *	7.64 ± 2.3	4.38 ± 2.03	3.64 ± 1.78	3.09 ± 1.17	0.019	I IV, II III
Pi10(mm)*	3.97 ± 0.05	3.96 ± 0.07	3.96 ± 0.05	4.01 ± 0.05	0.026	I II III, IV

* non-normally distributed data which were used Kruskal–Wallis Test; *p* value: One-way ANOVA analysis.

## Data Availability

The data presented in this study are available on request from the corresponding author. The data are not publicly available due to privacy.
